# Complete Genome Sequence of *Mycobacterium abscessus* subsp. *bolletii*

**DOI:** 10.1128/genomeA.00543-16

**Published:** 2016-06-09

**Authors:** Lindsay J. Caverly, Theodore Spilker, John J. LiPuma

**Affiliations:** Department of Pediatrics, University of Michigan Medical School, Ann Arbor, Michigan, USA

## Abstract

We report the complete genome sequence of a *Mycobacterium abscessus* subsp. *bolletii* isolate recovered from a sputum culture from an individual with cystic fibrosis. This sequence is the first completed whole-genome sequence of *M. abscessus* subsp. *bolletii* and adds value to studies of *M. abscessus* complex genomics.

## GENOME ANNOUNCEMENT

The *Mycobacterium abscessus* complex consists of three subspecies of rapidly growing mycobacteria (*M. abscessus* subsp. *abscessus*, *M. abscessus* subsp. *massiliense*, and *M. abscessus* subsp. *bolletii*) that are respiratory pathogens of humans (1, 2). Distinguishing between these subspecies may have implications for *M. abscessus* complex epidemiology, infection, disease course, and treatment response in individuals with cystic fibrosis and other lung diseases (2–4). Here, we report the complete genome sequence of FLAC003, an *M. abscessus* subsp. *bolletii* strain, isolated from the sputum of an individual with cystic fibrosis*.* This represents the first completed whole-genome sequence of *M. abscessus* subsp. *bolletii.*

Bacteria were grown on Middlebrook 7H11 agar at 37°C. The bacterial culture was resuspended in 1 ml of ultrapure water and adjusted to an optical density of 0.45, corresponding to approximately 10^9^ CFU. Genomic DNA was extracted from 400 µl of the adjusted suspension using the MagNA Pure Compact nucleic acid isolation kit (Roche, Indianapolis, IN, USA) following the manufacturer’s instructions. Genomic DNA libraries were prepared using an Illumina TruSEQ DNA library kit and sequenced on an Illumina HiSEQ 4000 paired-end flow cell (2 × 150-bp read length, V4 Chemistry) at the University of Michigan Medical School DNA Sequencing Core. Output files containing FASTQ reads were checked and edited using Trimmomatic version 0.33 (5). Read correction and assembly of the draft genomes was carried out using SPAdes version 3.5.0 (6).

The draft genome of FLAC003 was aligned with the 12 completed and annotated *M. abscessus* complex genomes currently available in NCBI and the draft genome of the *M. abscessus* subsp. *bolletii* type strain BD^T^ (7, 8) (http://www.ncbi.nlm.nih.gov/genome/browse/) using the Parsnp and Gingr programs of the Harvest tools version 1.2 suite (9). All 12 of the previously completed *M. abscessus* complex genomes (seven of which are designated *M. abscessus* subsp. *bolletii* in NCBI) clustered with either *M. abscessus* subsp. *abscessus* type strain ATCC 19977^T^ (10) or *M. massiliense* strain CCUG 48898 (11, 12). The draft genome of FLAC003 clustered with *M. abscessus* subsp. *bolletii* strain BD^T^ (7, 8).

The 22 contigs of *M. abscessus* subsp. *bolletii* strain BD^T^ were ordered using the completed genome of *M. abscessus* ATCC 19977^T^ as a reference with Mauve version 2.4.0 (13). The strain BD^T^ was then used to order the 25 contigs of FLAC003 with Mauve version 2.4.0. The sorted draft genome of FLAC003 was manually gap filled by identifying short segments (15 to 25 bp) on the ends of two contiguous pieces that matched to both ends of a single contig within FLAC003 not already included by Mauve in the alignment. These matches were verified by obtaining the longest possible perfect match on both sets of ends, checked with BLASTN for continuity, confirmed with BLASTX when possible, and checked for the appropriateness of gap distance against strain BD^T^. The FLAC003 genome was annotated using NCBI’s whole-genome shotgun submission portal containing the automated Prokaryotic Genomic Annotation Pipeline (PGAP) option. The complete genome is 4,826,045 bp in length and contains 4,606 proteins.

### Nucleotide sequence accession number.

This whole-genome shotgun project has been deposited in GenBank under the accession number CP014950.
